# Chatbots That Deliver Contraceptive Support: Systematic Review

**DOI:** 10.2196/46758

**Published:** 2024-02-27

**Authors:** Rhiana Mills, Emily Rose Mangone, Neal Lesh, Gayatri Jayal, Diwakar Mohan, Paula Baraitser

**Affiliations:** 1 SH24 London United Kingdom; 2 The Bill & Melinda Gates Foundation Seattle, WA United States; 3 Dimagi Cambridge, MA United States; 4 Department of International Health Johns Hopkins Bloomberg School of Public Health Baltimore, MD United States

**Keywords:** chatbot, contraceptives, digital health, AI, systematic review, conversational agent, development best practices, development, counseling, communication, user feedback, users, feedback, attitudes, behavior

## Abstract

**Background:**

A chatbot is a computer program that is designed to simulate conversation with humans. Chatbots may offer rapid, responsive, and private contraceptive information; counseling; and linkages to products and services, which could improve contraceptive knowledge, attitudes, and behaviors.

**Objective:**

This review aimed to systematically collate and interpret evidence to determine whether and how chatbots improve contraceptive knowledge, attitudes, and behaviors. Contraceptive knowledge, attitudes, and behaviors include access to contraceptive information, understanding of contraceptive information, access to contraceptive services, contraceptive uptake, contraceptive continuation, and contraceptive communication or negotiation skills. A secondary aim of the review is to identify and summarize best practice recommendations for chatbot development to improve contraceptive outcomes, including the cost-effectiveness of chatbots where evidence is available.

**Methods:**

We systematically searched peer-reviewed and gray literature (2010-2022) for papers that evaluated chatbots offering contraceptive information and services. Sources were included if they featured a chatbot and addressed an element of contraception, for example, uptake of hormonal contraceptives. Literature was assessed for methodological quality using appropriate quality assessment tools. Data were extracted from the included sources using a data extraction framework. A narrative synthesis approach was used to collate qualitative evidence as quantitative evidence was too sparse for a quantitative synthesis to be carried out.

**Results:**

We identified 15 sources, including 8 original research papers and 7 gray literature papers. These sources included 16 unique chatbots. This review found the following evidence on the impact and efficacy of chatbots: a large, robust randomized controlled trial suggests that chatbots have no effect on intention to use contraception; a small, uncontrolled cohort study suggests increased uptake of contraception among adolescent girls; and a development report, using poor-quality methods, suggests no impact on improved access to services. There is also poor-quality evidence to suggest increased contraceptive knowledge from interacting with chatbot content. User engagement was mixed, with some chatbots reaching wide audiences and others reaching very small audiences. User feedback suggests that chatbots may be experienced as acceptable, convenient, anonymous, and private, but also as incompetent, inconvenient, and unsympathetic. The best practice guidance on the development of chatbots to improve contraceptive knowledge, attitudes, and behaviors is consistent with that in the literature on chatbots in other health care fields.

**Conclusions:**

We found limited and conflicting evidence on chatbots to improve contraceptive knowledge, attitudes, and behaviors. Further research that examines the impact of chatbot interventions in comparison with alternative technologies, acknowledges the varied and changing nature of chatbot interventions, and seeks to identify key features associated with improved contraceptive outcomes is needed. The limitations of this review include the limited evidence available on this topic, the lack of formal evaluation of chatbots in this field, and the lack of standardized definition of what a chatbot is.

## Introduction

Recent guidelines have identified digital technologies as “promising” for improving knowledge; influencing attitudes, beliefs, and expectations; and increasing self-efficacy in support of healthy sexual and reproductive health (SRH) behaviors [1-3]. Although these guidelines consider digital interventions in general, there is a growing interest in building a stronger evidence base on how SRH programs might leverage key technologies to improve their reach, engagement, and impact. One technology that has seen a substantial rise in use by SRH programs is chatbots [4,5].

A chatbot is a computer program that is designed to simulate conversation with humans [6]. Chatbots may use rule-based language applications or more advanced natural language processing (NLP) and artificial intelligence technology to automate conversations [7]. In the context of contraception, chatbots have the potential to provide rapid and responsive information, counseling, and linkages to products and services [8]. They may also serve as navigational agents and companions on a reproductive health journey or be gamified and serve as “edutainment” [9].

There is a small but expanding literature within health care on chatbot design, user experience, and the outcomes of chatbot use. For example, within mental health care, there have been several recent reviews on chatbot design [10,11], chatbots that enable service delivery [12], and the ability of chatbots to build relationships with humans [6,13]. There has been no attempt, to date, to consolidate and synthesize the literature on chatbots to improve contraceptive outcomes or to inform best practices for the design and development of chatbots for this purpose.

## Methods

### Objectives

This review aims to systematically collate and interpret evidence to determine whether and how chatbots improve contraceptive knowledge, attitudes, and behaviors. Contraceptive knowledge, attitudes, and behaviors include access to contraceptive information, understanding of contraceptive information, access to contraceptive services, contraceptive uptake, contraceptive continuation, and contraceptive communication or negotiation skills. A secondary aim of the review is to identify and summarize best practice recommendations for chatbot development to improve contraceptive outcomes including the cost-effectiveness of chatbots, where evidence is available.

To achieve these aims a PICO (Population, Intervention, Control, Outcome) framework was used to identify the components that should be included in the review process (Textbox 1).

**PICO framework for systematic review.**

**Population**

- Users of an existing chatbot in any global context

**Intervention**

- Chatbots that improve access to contraceptive information, understanding of contraceptive information, access to contraceptive services, contraceptive uptake, contraceptive continuation, and contraceptive communication or negotiation skills

**Control**

- Any alternative intervention or no intervention

**Outcomes**

- Improved contraceptive knowledge, attitudes, or behaviors or best practice recommendations for developing chatbots to deliver these outcomes in an effective or cost-effective manner

### Search Strategy

We systematically searched and collated peer-reviewed, original research, and gray literature sources published between January 2010 and September 2022, which reported outcome data and data collection methods. We chose to include gray literature because we anticipated very little published evidence available on chatbots that address contraceptive outcomes, as this technology is relatively new.

Searches were carried out in the following databases in October 2022: MEDLINE, Embase, EmCare, PubMed, Science Direct, Cochrane Library, Scopus, and Google Scholar. The search terms detailed in Textbox 2 were used to search the selected databases (see Multimedia Appendix 1). A search for gray literature was completed using the Google search engine using the following search terms: “chat bot” OR chatbot OR “conversational agent” AND contracept* OR “family planning.” The first 100 Google search hits were screened.

Sources identified from the database and Google searches were imported into reference management software. Duplicates were removed, and the title and abstract of each source were screened against the inclusion and exclusion criteria detailed in Textbox 3 by RM. After this initial screening, a secondary screening of full-text resources was undertaken, again using the inclusion and exclusion criteria in Textbox 2. RM screened the sources, and PB screened 10% of the sources with any disagreements discussed to check for accuracy and consistency.

**Search terms.**

**Chatbot terms**

- “chat bot*” or chat-bot* or chatbot* or “chatter bot*” or chatterbot* or “talk bot*” or talkbot* or talk-bot* or “interactive agent*” or “conversational agent*” or “artificial conversation* entit*” or “artificial intelligence” or AI or “human computer interaction” or “intelligent agent*” or “chat agent*” or “relational agent*” or “virtual agent*” or “virtual assistant*” or “virtual coach”

**Sexual and reproductive health terms**

- “sexual and reproductive health” or “reproductive and sexual health” or “sexual health” or “reproductive health” or “sexually transmitted infection*” or STI or STIs or “sexually transmitted disease” or STD or STDs or HIV of “human immunodeficiency virus” or chlamydia or gonorrhea or herpes or “herpes genitalis” or HPV or “human papillomavirus” or syphilis or condom or “cervical cancer” or “cervical screen” or “pap* test” or antenatal or prenatal or postnatal or perinatal or pregnan* or maternal or gynae* or birth or caesarean

**Contraceptive terms**

- Contracept* or “family planning” or LARC or “long acting reversible contraceptive” or “pill” or COC or POP or “progesterone only pill” or “combined oral contraception” or “inter-uterine device” or IUD or “inter-uterine system” or IUS or coil or “hormonal coil” or “copper coil” or “contracept* implant” or “injectable contracept*” or “self-injectable contracept* or “depo-provera” or “sayana Press” or “contraceptive decision making” or “family planning decision making”

**Inclusion and exclusion criteria.**

**Inclusion criteria**

- The paper must be published between 2010 and September 2022
- The intervention must include an existing chatbot
- The intervention must aim to address an element of contraception—this need not be the main focus, but it must be included

**Exclusion criteria**

- The paper is not published between 2010 and September 2022
- The intervention does not include an existing chatbot
- The intervention does not aim to address an element of contraception
- The paper is not of reasonable research quality standard

### Quality Assessment

**Figure 1:**
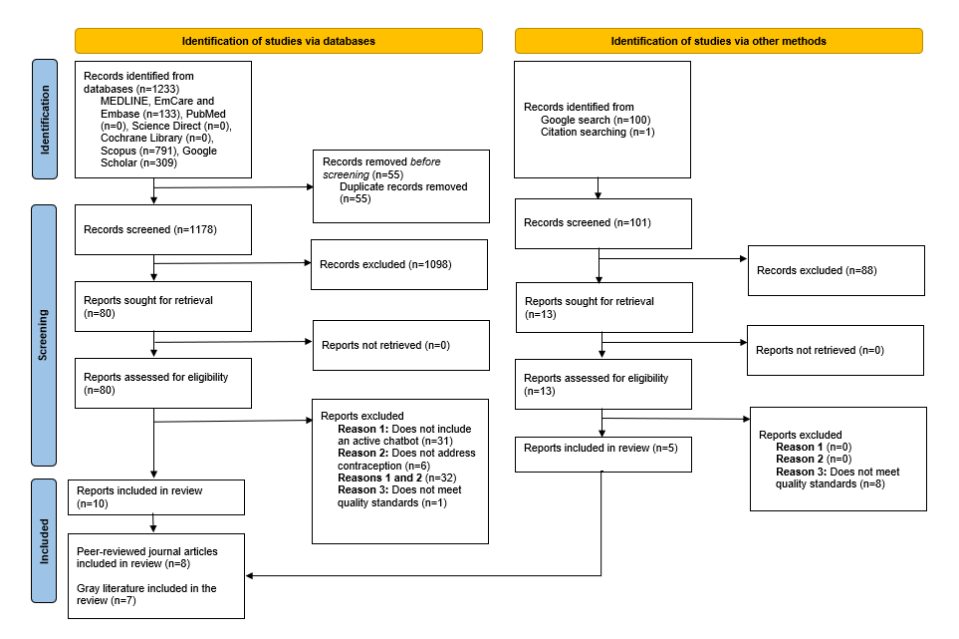
PRISMA (Preferred Reporting Items for Systematic reviews and Meta-Analyses) 2020 flow diagram for new systematic reviews that included searches of databases, registers, and other sources.

Sources were critically appraised for research and reporting quality. The Critical Appraisal Skills Programme (CASP) Cohort Study appraisal tool, the CASP Qualitative Study appraisal tool, the CASP Randomized Control Trial (RCT) Study appraisal tool, and the AACODS (Authority, Accuracy, Coverage, Objectivity, Date, and Significance) checklist for gray literature were used to guide the critical appraisal process [14,15]. CASP tools use criteria to assess how methodologically sound a source is, how accurately and comprehensively results have been reported, and whether ethical considerations have been accounted for. The AACODS tool assesses the rigor and relevance of gray literature sources. PB appraised a randomly selected sample of the peer-reviewed journal articles to check for agreement on quality assessment. The authors RM, ERM, NL, GJ, and PB critically discussed the quality assessment of gray literature papers. A total of 9 sources were excluded because of not meeting quality standards (see Figure 1).

### Data Extraction

Data were extracted from the included sources by RM using a standard data extraction template, which allowed for a standard set of variables to be extracted from each source (see Multimedia Appendix 2). We noted that standard variables were not reported in a source. Verbatim sections of text, which described best practice recommendations, were separately extracted from the sources that described the design and development and user feedback on chatbots and added to a spreadsheet. RM and PB regularly discussed data extraction to check for accuracy.

### Synthesis

The data extracted from all sources were grouped under data extraction categories: use of theory to underpin chatbot development; efficacy and impact; improving knowledge; user engagement; number of users; length of engagement; demographics of those who engaged; user feedback; acceptability; convenience and accessibility; and anonymity, nonjudgment, and privacy.

Because of insufficient quantitative evidence for meta-analysis, quantitative findings were summarized under appropriate data extraction headings and are reported in summary in the Results section.

A narrative synthesis approach was used to collate and summarize qualitative evidence under each data extraction heading. The data under each heading were read and reread by RM to identify emerging themes and subthemes. Themes were considered emergent when multiple data points could be grouped in a theme or subtheme. RM regularly presented emergent themes and discussed the synthesis process with PB to check for accuracy and collaboratively develop themes. The synthesis process was shared with the authorship team on 2 occasions to check for accuracy.

Thematic analysis was also used to draw out best practices from the extracted data. RM grouped emerging themes that provided pragmatic information regarding best practice for chatbot development. These grouped themes were discussed with the authorship team to check for analytic accuracy and collaboratively label the best practices. Best practice guidance was only included if it was referenced across more than 1 evidence source.

## Results

### Overview

The PRISMA (Preferred Reporting Items for Systematic reviews and Meta-Analyses) diagram (Figure 1) summarizes the results of the search process. Although we used a broader search strategy with regard to date, to identify all literature in this under-researched field, we did not find any literature that predated 2018. Only 15 sources were identified for evaluation, reflecting the nascent development of chatbots in this field.

### Quality Assessment

The quality assessment process excluded 8 gray literature sources and 1 peer-reviewed journal article. The 8 gray literature sources were excluded based on the AACODS checklist, which measures the authority of the source author and the content of the source in terms of accuracy, coverage, objectivity, date, and significance. Three sources were excluded because they did not come from a reputable source and lacked accuracy in the methods they used to evaluate chatbots and reporting of their methods (where methods were reported at all). Five sources were excluded because they lacked accuracy in their methods and reporting and because the content of the source was not considered significant. For example, some sources were website articles that described a chatbot for a lay audience and included very little or no detail on the evaluation of the chatbot or outcomes of its use.

The single peer-reviewed journal article that was excluded had a qualitative study design and was excluded because of unclear research aims and sparse description of data analysis, meaning analytic rigor could not be ascertained.

### The Chatbots Identified

This review identified 15 sources, including 8 original research papers and 7 gray literature papers (see Tables 1 and 2). Seven chatbots in the review were implemented in high-income countries: the United Kingdom (n=2), United States (n=4), and Japan (n=1). Eight chatbots were implemented in low- or middle-income countries: Kenya (n=3), South Africa (n=1), Uganda (n=1), Nigeria (n=1), Bangladesh (n=1), and India (n=1). One chatbot had international reach. A total of 4 chatbots aimed specifically to engage the general population, and 6 sought to engage women and girls. Two of these specifically targeted young women: one targeted adolescent girls and one targeted young pregnant women. Three chatbots were aimed at adolescents of any gender. One chatbot was aimed at married couples, and one targeted those at risk of poor sexual health.

**Table 1:** Table of results.

Study	Date	Title	Source type	Source country	Study design	Chatbot name
Bonnevie et al [16]	2021	Layla’s Got You: developing a tailored contraception chatbot for Black and Hispanic young women	Peer-reviewed journal article	United States	Development report	Layla’s Got You
Brannock et al [17]	2019	Investigating attitudes and preferences towards a chatbot pregnancy guide within Facebook’s social media platform amongst pregnant women in Kenya	Gray literature: thesis	Kenya	Development report	Mama Support
Chernick et al [18]	2021	The perspectives of women and their health-care providers regarding using an ECA to support mode of birth decisions	Peer-reviewed journal article	United States	Qualitative study	Dr Erica
Green et al [19]	2022	Predicting health care-seeking behavior based on stated readiness to act: development and validation of a prediction model.	Peer-reviewed journal article	Kenya	Model development report	Ask Nivi
Handforth and Bertermann [20]	2018	How Girl Effect built a chatbot	Gray literature: technical report	South Africa	Development report	Big Sis
Hussain et al [21]	2019	Mobile phone-based chatbot for family planning and contraceptive information	Gray literature: conference paper	United States	Feasibility study	Name not reported
Woo et al [22]	2020	Development of conversational artificial intelligence for pandemic healthcare query support	Peer-reviewed journal article	United Kingdom	Development report	Akira
Maeda et al [23]	2020	Promoting fertility awareness and preconception health using a chatbot: a randomized controlled trial	Peer-reviewed journal article	Japan	RCT^a^	Name not reported
Nadarzynski et al [24]	2021	Barriers and facilitators to engagement with artificial intelligence (AI)-based chatbots for sexual and reproductive health advice: a qualitative analysis	Peer-reviewed journal article	United Kingdom	Qualitative interview study	Pat
Population Services International [25]	2019	Will access to sex-positive and reproductive health information through a chatbot lead to increased contraceptive use amongst Kenyan youth?	Website article	Kenya	Development report	Pleasure Bot
Rahman et al [26]	2021	AdolescentBot: Understanding opportunities for chatbots in combating adolescent sexual and reproductive health problems in Bangladesh	Peer-reviewed journal article	Bangladesh	Development report	AdolescentBot
Wang et al [27]	2022	An artificial intelligence chatbot for young people’s sexual and reproductive health in India (SnehAI): instrumental case study	Peer-reviewed journal article	India	Instrumental case study	SnehAI
Wilson et al [28]	2017	In bed with Siri and Google Assistant: a comparison of sexual health advice	Gray literature: short report	Not context specific	Short report	Siri and Google Assistant
Winskell et al [29]	2021	Building a chatbot for health content? This is for you.	Website article	Nigeria and Uganda	N/A^b^	Tina
Work & Co [30]	2022	Planned parenthood case study	Website article	United States	Development report	Ask Roo

^a^RCT: randomized controlled trial.

^b^N/A: not applicable.

**Table 2:** Table of results, continued.

Study	Chatbot aim	Chatbot target group	Chatbot platform	Method of input by user	Chatbot persona	How does the chatbot address contraception
Bonnevie et al [16]	SRH^a^ information dissemination	Black and Hispanic women in Onondaga County, New York	Website	Free text input	Female peer	The chatbot includes content on contraception, as part of wider SRH content
Brannock et al [17]	Pregnancy information dissemination	Pregnant women	Facebook Messenger	Menu selection input	Not reported	The chatbot includes content on postnatal contraception, as part of wider content on pregnancy
Chernick et al [18]	Contraception information dissemination	Adolescent girls presenting at an emergency department	SMS	Menu selection input	Health care worker	The chatbot disseminates information on contraception
Green et al [19]	SRH information dissemination and linkage to SRH services	General population	WhatsApp	Menu selection input	No persona	The chatbot includes content on SRH as part of wider SRH content, including contraception decision-making
Handforth and Bertermann [20]	SRH information dissemination and linkage to SRH services	Young women	WhatsApp	Menu selection input	Female relative	The chatbot includes content on contraception as part of wider SRH content
Hussain et al [21]	Contraception information dissemination	Married couples	SMS	Menu selection input	No persona	The chatbot disseminates information on contraception
Woo et al [22]	Pandemic setting health information dissemination	General population	Not reported	Free text input	Knowledgeable young woman	The chatbot includes content on contraception, as part of wider SRH content
Maeda et al [23]	Preconception care and fertility information dissemination	Women	Not reported	Free text input	Not reported	The chatbot includes content on oral contraceptives, as part of wider fertility and preconception care content
Nadarzynski et al [24]	SRH information dissemination	Those at higher risk of poor SRH	Website	Free text input	Neutral robot	The chatbot includes content on contraception, as part of wider SRH content
Population Services International [25]	Pleasure positive SRH information dissemination	General population	WhatsApp	Menu selection input	Knowledgeable young woman	The chatbot includes content on contraception, as part of wider SRH content
Rahman et al [26]	SRH information dissemination	Adolescents	Facebook Messenger	Free text input	Neutral robot	The chatbot includes content on contraception, as part of wider SRH content
Wang et al [27]	SRH information dissemination	Adolescents	Facebook Messenger	Free text input	Health care worker	The chatbot includes content on contraception, as part of wider SRH content
Wilson et al [27]	Information dissemination and digital personal assistant	General population	Google, Google Assistant, Siri	Free speech input	Knowledgeable young woman	The chatbot can answer SRH queries, as part of wider content
Winskell et al [29]	SRH information dissemination	Young women	WhatsApp	Menu selection input	Female peer	The chatbot can answer SRH queries, as part of wider content
Work & Co [30]	SRH information dissemination	Adolescents	Website	Free text input	Neutral robot	The chatbot includes content on contraception, as part of wider SRH content

^a^SRH: sexual and reproductive health.

Three sources focused solely on improving contraceptive outcomes. The Dr Erica chatbot aimed to improve contraceptive knowledge and uptake among adolescent girls presenting at an emergency department in the United States [18]. An analysis of the AskNivi chatbot in Kenya aimed to predict SRH service use after seeking contraceptive information from a chatbot [19]. Finally, a feasibility study evaluated a mobile phone chatbot providing contraceptive information for couples in the United States [21].

The majority of sources identified (n=11) aimed to improve SRH knowledge, attitudes, or behaviors, and contraceptive outcomes were a subset of these [16,17,20,23-30]. One chatbot answered health queries in a COVID-19 pandemic setting, including queries regarding contraception [22].

The chatbots described in our sources had a range of personas. Two had a female peer persona [16,29], 1 had a female relative persona [20], 3 had robot personas that were neutral in age and gender, 4 had knowledgeable female personas [22,25,28], 2 had female health care worker personas [18,27], 2 did not have a persona, and 2 sources did not report on the persona.

Seven of the chatbots in our sample allowed for free text to be inputted by the user and 2 allowed for free speech input. These chatbots use NLP to interpret the user’s input, but none of the chatbots in our sample used NLP to generate responses. Seven of the chatbots in our sample used menus of queries and responses to allow users to interact with the chatbot.

Chatbots can be hosted on a range of platforms. In this sample, chatbots were hosted on a website (n=3), WhatsApp (n=4), and Facebook Messenger (n=3), and 2 interacted with the user via SMS text messages. Two chatbots were devices that responded to free speech input. Two sources did not report platform.

### Use of Theory to Underpin Chatbot Development

Only 3 sources used a theoretical framework to underpin their analysis. The development of a contraceptive care-seeking predictive model used a “stages of change” model to predict whether women were “ready to act” to access contraceptives [19]. A case study of a national chatbot for adolescents applied Gibson’s “theory of affordances,” which seeks to understand how relational dynamics between users and environments can enable or prohibit certain actions [27]. In an analysis of an SMS text messaging chatbot, the Unified Theory of Acceptance and Use of Technology model was used to understand how effort, attitude, and user expectations impact behavioral intentions to use a chatbot [21].

### Efficacy and Impact

There is little evidence to assess the effectiveness of chatbots in impacting contraceptive knowledge, attitudes, and behaviors. We identified 1 RCT, 1 small cohort study, 1 predictive model development report, 2 development reports, and 1 gray literature report on chatbot impacts on contraceptive behaviors. We found 1 informal report on the chatbot impact on contraceptive knowledge and no evidence on the impact on contraceptive attitudes.

The RCT recruited women planning a pregnancy in Japan and assessed effectiveness using pre- and postintervention surveys on fertility knowledge and behaviors including intention to use oral contraceptives (OCs) after birth [23]. In this double-blinded RCT, participants (n=927) were randomized into 3 groups. The intervention group interacted with the chatbot, control group 1 was invited to read a PDF containing the same fertility and preconception care information, and control group 2 was instructed to read a PDF about the National Pension System. Intention to use OCs was significantly higher in the intervention group than control group 2 (*P*<.001) but significantly lower in the intervention group compared with control group 1 (*P*=.005), suggesting that the chatbot did not impact on intention to use OCs compared with a leaflet with the same content [23].

The small cohort study [18] measured the uptake of hormonal contraception after interaction with the Dr Erica chatbot, an SMS text messaging chatbot providing contraception information. A convenience sample of adolescent girls (n=42) were recruited in an emergency department in the United States and received messages from Dr Erica over 10 weeks inviting the discussion of contraception. There was no control group. Of the 35 participants who completed follow-up through a web-based or telephone survey, 16 (46%) initiated hormonal contraceptives. Of the 30 participants who completed follow-up and were sexually active in the previous 3 months, 52% (15/29) had initiated hormonal contraceptives and 66% (20/30) had used any contraceptive at last intercourse. None of the participants became pregnant [18].

Other evidence on the efficacy of chatbots for contraceptive behavior outcomes is found in the evaluation of the Population Services International (PSI) chatbot in Kenya [25]. PSI piloted a chatbot that disseminated pleasure-positive SRH information including contraceptive information with 1000 referral vouchers for local SRH services issued to those who expressed interest in and intent to use these services. The study found that none of the vouchers were redeemed [25].

A third study reports on data from the users of contraceptive chatbot content within the AskNivi chatbot and predicted that 29% (233/817) of female users were “ready to act” to access contraceptive services [19]. Within 2 weeks of interaction with the chatbot, the chatbot sent up to 2 “check-in” messages and asked users to self-report whether they had sought contraceptive services. In total, 93% (760/817) of women classified by the model as “ready to act” reported accessing contraceptive services. The model was internally validated with a test set of AskNivi users. The model was found to slightly overpredict readiness to act [19].

### Improving Knowledge

The only evidence found on the impact of chatbots on contraceptive knowledge comes from a technical report [20] on the development of a chatbot, Big Sis, in Kenya. This report includes the testing of user knowledge before and after engagement with an educational quiz within the chatbot. Average knowledge scores among users (n=419) improved by 36% (*P*<.001) after engaging with chatbot content. Big Sis users were also asked to complete a survey (11% response rate) through which they reported increased awareness and understanding of contraception. No comparison group is reported [20].

One source reports on the accuracy of SRH information provided by voice-activated and response chatbots [19]. This source compared 2 voice-activated and response chatbots (Google Assistant and Siri) for accuracy and relevance against written Google searches using 50 SRH queries. Google searches outperformed both chatbots, providing 72% (36/50) of the best or equal best responses. Google searches also had the lowest outright failure rate, returning no completely inaccurate or irrelevant answers. Google Assistant performed better than Siri with 50% (25/50) of best or equal best responses versus 32% (16/50); *P*=.04 [19].

### User Engagement

Eight sources report user engagement data through number of users, messages received, length of engagement, returning users, length of interaction, number of interactions with web-based promotional material, and the demographics of those who engaged.

### Number of Users

Two chatbots reached large audiences, with SnehAI reaching 135,263 unique chatbot users who exchanged 8,170,879 messages with the chatbot over a 5-month period [27] and Ask Roo reporting 3.5 million conversations with users over a year but no data on number of unique users [30]. Other chatbots reached smaller audiences, with Tina reaching 7000 users within a 6-month period [29], Layla’s Got You receiving 6164 messages over 21 months [16], and PSI’s pleasure-positive chatbot reaching 4866 users over an unspecified time period [25]. Over 10 weeks, 83% (35/42) of Dr Erica users interacted with at least 1 message sent by the chatbot [18].

The number of people interacting with web-based promotional material is reported from the Leyla’s Got You chatbot, which shows that a promotional campaign hosted across social media accounts had 2,483,683 impressions [16]. The web-based promotional campaign for Tina had 300,000 click-throughs to find out more about self-injectable contraceptives [29], and Big Sis web-based promotion recruited more than 2500 users for testing purposes [20].

### Length of Engagement

Users engaged with SnehAI on average for 1.9 sessions for 7.6 minutes and exchanged 56.2 messages with the chatbot [27]. In total, 59% (1632/2767) of Big Sis users interacted with the chatbot for less than 1 hour, and reengagement rates were 23% to 43% after reminder messages [20]. PSI reports that 1866 users returned to the chatbot [25], with click-throughs to 2 or more branches of the chatbot content increasing from 67% to 83% with the introduction of pleasure content [25]. A Facebook Messenger chatbot, Mama Support, reported that in a 4-week period, a sample of 22 users interacted with the chatbot on average 19 times after reminders were sent [17].

### The Demographics of Those Who Engaged

SnehAI engaged users who self-reported as 93% (125,795/135,263) male and 6.8% (9198/135,263) female, with the authors explaining this through the high levels of stigma and shame experienced by young women in this context around engaging with SRH content [27]. Ask Roo reports that 78% of its users are 13 to 19 years old, suggesting that they reached their target audience [30].

### User Feedback

A total of 6 studies report user feedback on chatbots. Qualitative interviews were carried out with 40 participants who were at higher risk of poorer sexual health, aged between 18 and 50 years in the United Kingdom, and interacted with the Pat chatbot [24]. Interviews were also carried out with the users of Mama Support (n=22), who were pregnant women aged between 25 and 35 years in urban areas of Kenya [17]. A web-based survey of 256 participants was gathered from the users of AdolescentBot, who were adolescents aged between 10 and 19 years in Bangladesh [26]. Participants (n=278), women aged between 20 and 34 years in Japan, who interacted with the preconception care chatbot provided written feedback on chatbot use [23]. User feedback for Dr Erica was collected at follow-up via a web-based survey or telephone survey with female emergency department patients aged between 14 and 19 years in the United States [18]. The users of Akira (n=57), the pandemic health query chatbot, gave feedback through a user satisfaction survey and were aged between 18 and 20 years in the United Kingdom [22].

### Acceptability

A wide range of users found chatbots acceptable. Adolescent girls found that Dr Erica was acceptable, with 94% (29/31) of participants liking the SMS text messages they interacted with and 83% (25/30) stating that they would recommend the chatbot to a friend [18]. Akira received an average satisfaction rating of 2.74 out of 5, although satisfaction decreased with each query that users asked [22]. Pregnant women who used Mama Support(n=22) said that they would refer the chatbot to a friend [17].

Users across different groups experienced chatbots as trustworthy [17,26]. Adolescents found chatbots trustworthy when the chatbots seemed to give accurate answers to their questions [26], and pregnant women trusted a chatbot when it was associated with a reputable organization [17]. Users experienced chatbots as incompetent because either the chatbots could not answer their questions or they had experienced technical difficulties with the platform or device [17,23,24]. Where chatbot content was limited to SRH, some users reported that they wanted to ask questions outside of these limits [17,26]. For example, some adolescents wanted AdolescentBot to provide other health content such as self-care tips for the common cold [26].

Feedback on chatbots included negative experiences of the chatbot’s lack of humanness across user groups, including lack of empathy, limited interaction, and experience of the chatbot as “cold” and robot-like [23,24].

### Convenience and Accessibility

A wide range of users felt that chatbots were convenient to use because they could be accessed at any time and from any location [23,24,26]. They valued fast and accurate responses to queries that did not require searching the internet, appraising internet sources, or reading long passages of text [17,24]. However, women in Japan felt that interacting with a chatbot was “burdensome” and that reading an accurate document would be more convenient [23]. The users of Pat thought that chatbots were convenient sign posters to local and appropriate services [24]. The users of Mama Support disliked asking questions through a menu and would prefer to ask their own questions via free text [17].

### Anonymity, Nonjudgment, and Privacy

A wide range of users appreciated the anonymity that talking to a chatbot afforded them [17,24,26], but some users had concerns about the privacy of the data provided to the chatbot [24]. Users suggested that they felt less shy talking to a chatbot and that chatbots were a good tool for those who would find it difficult to ask questions face to face because of embarrassment or stigma [17,23,24]. This was reported across user groups.

### Cost-Effectiveness

There was no evidence available on the costs or cost-effectiveness of chatbots for impacting contraceptive knowledge, attitudes, and behaviors.

### Best Practices

The secondary aim of this review was to establish best practice for developing chatbots to improve contraceptive outcomes. We reviewed all the evidence collected to identify best practice recommendations that came from more than 1 paper. A total of 7 best practices that emerged from the literature are listed in Table 3.

**Table 3:** Best practices.

Best practice	Quotation
It is best practice to design and develop chatbots with input from the target audience [16,20,29,30]	“The user experience is sacrosanct. All decisions need to be made with users at the center. This begins with understanding user needs and realities, including whether or not a chatbot is a channel with which they can engage” [20]
Chatbot use for contraceptive information is a new behavior; it is best practice for users to be given information on chatbots, how to use them, and why to use them before their first use [24,29]	“Using a chatbot to get information and support about health-related content (and specifically, sexual & reproductive health) is a completely new behavior. It isn’t enough to tell consumers WHERE to find your chatbot, you also need to educate them on WHAT a chatbot is and WHY they should use it – the value it offers them as a platform” [29]
Using a chatbot should be intuitive and easy (frictionless) with no superfluous steps to maintain user engagement and avoid drop-offs [20,29]	“Our reading behavior online is to dip in and out of content in an ‘on-demand’ fashion. Long and complex menus can be overwhelming and turn people off. If you have directed people to your chatbot with a very specific call-to-action, don’t make them work too hard to find the information they are interested in” [29]
Chatbots should be hosted on devices and platforms commonly used by the target audience, so that chatbot use is seamless with their routine digital behavior [20,26]	“27% of all users could not see Quick Reply buttons due to the application version or handset that they were using…These issues appeared to be most common amongst users with the Facebook Lite application, those using third-party applications, and those using older handsets. In response, we created an alternative to Quick Replies using numeric menus” [20]
Linking a chatbot with a reputable and well-known organization can build user trust [17,29]	“As I have said many times now, I saw that it [the chatbot] was related to Jacaranda Maternity. Of course, there are the reviews [of Jacaranda] and the reviews were so good. And I felt that by interacting with this chatbot, I was getting very relevant information from a very qualified source. So, I felt nothing fishy at all and I considered it a lot...” [FGD participant] [17]
The persona of a chatbot should resonate with the intended audience and reflect the intended relationship to the audience, for example, peer, health care worker, or neutral robot [16,20,22,25,30]	“A young woman of color was then hired as a copywriter to provide Layla with a ‘personality’ – rewriting responses using language that would resonate with the target audience” [16]
User privacy should be ensured and visible; this will increase user trust in the chatbot [20,24]	“I’m a bit worried especially due to data protection so I wouldn’t do it. If I didn’t have the certainty that my data protection absolutely complete itself so yes I don’t feel comfortable talking about my sexual health online” [Interview participant] [24]
Some access to human support is important for those in need of urgent help, especially where the intended audience is young people [20,27]	“Humans are still important!... During the test phase, we had 243 triggers, with 61 being interactions that we would classify as a safeguarding alert. These interactions would be addressed via human intervention to connect girls with suitable support services” [20]

## Discussion

### Principal Findings

The evidence available to assess whether and how chatbots improve contraceptive knowledge, attitudes, and behaviors is sparse and conflicting. One RCT suggests no effect on intention to use contraception [23]. This RCT is well designed and has a large overall sample size of 927, split evenly between an intervention and 2 appropriate control groups. This study relies on self-reporting of intention to use OC rather than uptake of OC. The sample also had a higher prevalence of OC use than national data at baseline. Overall, the methodological quality here is high, and this evidence is considered to be the strongest evidence available on the impact of chatbots on contraceptive outcomes. A cohort study suggests increased uptake of contraception in adolescent girls [18]. However, this cohort study has a small sample size of 42 and did not include a control group. Without a control group, it is difficult to ascertain whether the uptake of contraception in adolescent girls is due to interaction with the chatbot or due to other factors. A development report suggests no impact on access to services [25]. This was measured by counting the number of “referral vouchers” that were redeemed at local SRH services. This method of assessing the impact on access to services is suboptimal as users may have accessed the service but failed to redeem their voucher. There is poor-quality evidence to suggest increased contraceptive knowledge from interacting with chatbot content, as measured by in-chatbot quizzes [20]. This evidence is considered poor as, although the sample consists of 419 users, no control group was used. Also, this evidence measured user knowledge before and immediately after engagement with chatbot content. Longer-term knowledge retention is not measured. There are some concerns about the accuracy and relevance of the knowledge that personal assistant chatbots provide [28]. However, evidence from other fields suggests that chatbots could improve knowledge as they deliver information in a digestible and engaging format [31]. No evidence on the impact of chatbots on attitudes to contraception was found.

The chatbots included in this review showed limited user reach and engagement from over 100,000 users in 5 months [27] to under 10,000 in 6 months [29]. The reporting of engagement outcomes is highly variable, and the reporting of the marketing strategies used to drive engagement is limited, making interpretation of this evidence difficult.

The evidence base suggests that chatbots supporting contraceptive outcomes are acceptable, convenient, anonymous, and private, but that some users experience chatbots as incompetent, experience technical difficulties, find interaction burdensome, or experience chatbots as impersonal [17,18,23,24,26].

The best practice guidance from the contraception literature is consistent with that in the broader literature [6,10-13].

### Research Recommendations

Further research is needed to understand the impact of chatbot technologies on contraceptive outcomes, and it is important that this research consider the following issues: (1) Impact at scale: there are simply no studies that demonstrate that chatbots can improve contraceptive knowledge, attitudes, and behaviors at scale; whether chatbots are more effective among specific populations; and whether chatbots provide efficiencies in cost or value for money. (2) Most chatbot evaluations do not offer comparisons with alternative technologies. These comparisons are essential to understand the impact of the dynamic nature of content delivery (ie, the chatbot), which could also be provided through simpler means, such as printed materials or websites. (3) Where engagement is reported, some description of the strategies used to promote engagement, as well as the size of the target audience, is required to help with the interpretation of data on engagement. This is because engagement may be dependent on factors unrelated to chatbot acceptability, such as the level of promotion. (4) A classification of levels of interactivity within chatbots is required to understand their impact. For example, chatbots that ask users to choose from a menu of questions are very different from those that interpret free-text questions. Research that understands the relative value of more and less complex chatbots is required. (5) The late 2022 launch of generative AI chatbots like ChatGPT and subsequent iterations opens up new possibilities for engaging users and supporting their contraceptive needs. Large language bots are likely the wave of the future, but will need thoughtful evaluation to determine whether they can effectively and cost-effectively improve contraceptive knowledge, attitudes, and behaviors, and to carefully assess and mitigate associated risks.

### Limitations

First, we are aware of a number of chatbots that are active in the contraceptive space globally but have not been formally evaluated. To mitigate this, we conducted a thorough search of the gray literature, but it is still likely that this missed chatbots that have not been reported on at all and could therefore not be included in this review.

Second, a wide range of technologies are described as chatbots. These differ in their technical specifications, level of interactivity, method of response generation, target audiences, and the focus of their content. This makes collating the evidence challenging, and in this rapidly changing field, the published literature may not describe the most recent examples of this technology. This review used an up-to-date definition of a chatbot and included all technologies that met this definition regardless of whether they were described as a chatbot or otherwise.

Lastly, the lack of robust quantitative evaluations meant that it was not possible to complete a meta-analysis. Other methods of quantitative synthesis were explored, but ultimately the lack of evidence meant this was not possible. As more evidence becomes available in this field, a meta-analysis may be possible in future reviews.

### Conclusions

We found limited and conflicting evidence on chatbots to improve contraceptive knowledge, attitudes, and behavior. Further research should aim to understand the impact of chatbot interventions in comparison with alternative technologies, acknowledge the variation in chatbot interventions, and seek to identify key features associated with their impact.

## Appendix

Multimedia Appendix 1 Database search terms.

Multimedia Appendix 2 Data extraction framework.

Multimedia Appendix 3 PRISMA checklist.

## Abbreviations

AACODS: Authority, Accuracy, Coverage, Objectivity, Date, and Significance
CASP: Critical Appraisal Skills Programme
NLP: natural language processing
OC: oral contraceptive
PICO: Population, Intervention, Control, Outcome
PRISMA: Preferred Reporting Items for Systematic reviews and Meta-Analyses
PSI: Population Services International
RCT: randomized controlled trial
SRH: sexual and reproductive health

## Data Availability

The data sets generated during and/or analyzed during this study are available from the corresponding author on reasonable request.
