# Supplementary material for: Chatbots That Deliver Contraceptive Support: Systematic Review
Source: J Med Internet Res. 2024 Feb 27;26:e46758. doi: 10.2196/46758 (PMC10933731; doi:10.2196/46758)
Supplement: Multimedia Appendix 2 [file jmir_v26i1e46758_app2.docx]

**Multimedia Appendix 2**

Standardized data set extracted from each paper.

- Title
- Author
- Year
- Country
- Target population
- Inclusion of contraception
- Study design
- Sample size
- Intervention
- Control
- Theoretical framework
- Platform
- Persona
- Outcomes measured (general)
- Outcomes (general)
- Outcomes measured (contraception)
- Outcomes (contraception)
- User feedback (negative)
- User feedback (positive)
